# The 3′ Untranslated Regions of Influenza Genomic Sequences Are 5′PPP-Independent Ligands for RIG-I

**DOI:** 10.1371/journal.pone.0032661

**Published:** 2012-03-15

**Authors:** William G. Davis, J. Bradford Bowzard, Suresh D. Sharma, Mayim E. Wiens, Priya Ranjan, Shivaprakash Gangappa, Olga Stuchlik, Jan Pohl, Ruben O. Donis, Jacqueline M. Katz, Craig E. Cameron, Takashi Fujita, Suryaprakash Sambhara

**Affiliations:** 1 Influenza Division, National Center for Immunization and Respiratory Diseases, Centers for Disease Control and Prevention, Atlanta, Georgia, United States of America; 2 Pennsylvania State University, University Park, Pennsylvania, United States of America; 3 Division of Scientific Resources, National Center for Emerging and Zoonotic Infectious Diseases, Centers for Disease Control and Prevention, Atlanta, Georgia, United States of America; 4 Kyoto University, Kyoto, Japan; University of Georgia, United States of America

## Abstract

Retinoic acid inducible gene-I (RIG-I) is a key regulator of antiviral immunity. RIG-I is generally thought to be activated by ssRNA species containing a 5′-triphosphate (PPP) group or by unphosphorylated dsRNA up to ∼300 bp in length. However, it is not yet clear how changes in the length, nucleotide sequence, secondary structure, and 5′ end modification affect the abilities of these ligands to bind and activate RIG-I. To further investigate these parameters in the context of naturally occurring ligands, we examined RNA sequences derived from the 5′ and 3′ untranslated regions (UTR) of the influenza virus NS1 gene segment. As expected, RIG-I-dependent interferon-β (IFN-β) induction by sequences from the 5′ UTR of the influenza cRNA or its complement (26 nt in length) required the presence of a 5′PPP group. In contrast, activation of RIG-I by the 3′ UTR cRNA sequence or its complement (172 nt) exhibited only a partial 5′PPP-dependence, as capping the 5′ end or treatment with CIP showed a modest reduction in RIG-I activation. Furthermore, induction of IFN-β by a smaller, U/A-rich region within the 3′ UTR was completely 5′PPP-independent. Our findings demonstrated that RNA sequence, length, and secondary structure all contributed to whether or not the 5′PPP moiety is needed for interferon induction by RIG-I.

## Introduction

The innate immune system has evolved to recognize pathogen-associated molecular signatures leading to activation of innate immune receptors [1]. This activation results in the production of antiviral and proinflammatory cytokines that impair microbial replication and induction of adaptive immune responses that actively eliminate pathogens [2]. Different pathogen sensing receptors are found in multiple locations, such as in the cytosol, plasma and intracellular vesicular membranes, and extracellular tissue fluids, to better defend against microbes that have different metabolic requirements and tropism for cellular compartments [3], [4], [5], [6], [7], [8], [9]. Two cytosolic pathogen sensors, Retinoic Acid Inducible Gene-I (RIG-I) and Melanoma Differentiation-Associated gene-5 (MDA5) have been found to be critical in the activation of the type I interferon-dependant antiviral innate immune response [10], [11]. While RIG-I detects RNA species from a number of viruses belonging to paramyxoviridae, orthomyxoviridae, rhabdoviridae, filoviridiae and herpesviridae, MDA5 detects RNA primarily from picornaviruses [12]. However, both these sensors appear to detect at least some strains of West Nile virus [13]. Various ligands, including 5′PPP-ssRNA, short dsRNA, full-length genomes of RNA viruses, and poly-uridine motifs within 5′PPP genome termini have been reported to activate RIG-I [6], [14], [15], [16], [17], [18]. Since the effect of length, sequence, secondary structure, and 5′PPP of ssRNA on binding and activation have not been fully characterized, we investigated these structural features using several vRNA and cRNA species generated from the 5′ and 3′ UTR of influenza virus NS1 gene segment by *in vitro* transcription (IVT). Our findings indicated that RNA sequences found within the NS1 segment of the influenza viral genome were capable of inducing IFN-β *in vitro* based on their specific sequences and structures. In addition, U/A-rich elements within the genome had the ability to induce IFN-β in a 5′PPP-independent manner. Our findings have demonstrated that RIG-I has evolved to interact with multiple U/A-rich RNA motifs commonly found in the UTRs of many diverse RNA viruses, thus facilitating its role as a key pathogen sensor against a broad range of viruses.

## Results

### Nucleotide sequence-dependent interferon induction

**Figure 1:**
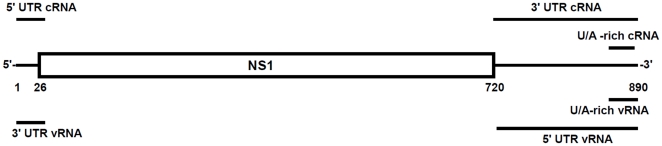
Schematic representation of RNAs used in this study. The influenza A virus segment 8 cRNA is shown with NS1 and NS2/NEP coding sequences boxed. The extended lines represent the 5′ and 3′ non-coding sequences. Bars (not drawn to scale) indicate sequences (see Materials and Methods) used to generate *in vitro* transcribed (IVT) RNAs.

The ends of the eight influenza A virus genomic RNA segments are highly conserved but only the shorter segments appear to provide optimal substrates for recognition by RIG-I [17]. Although the importance of the 5′PPP group for RIG-I binding is well established, the role of additional ligand characteristics is less clear. To investigate the potential contributions of the nucleotide sequence and structure of these regions to the activation of the innate immune system, we designed and examined several derivatives of the termini of genomic and viral RNAs from the shortest influenza A virus segment. These small influenza-derived ribonucleotide sequences were made by IVT and were used either with the naturally occurring 5′PPP modification intact, or without the 5′PPP, which was removed with CIP treatment or replaced with a cap analog. The sequence and the predicted secondary structure of the RNA species used in this study are shown in Fig. 1.

**Figure 2:**
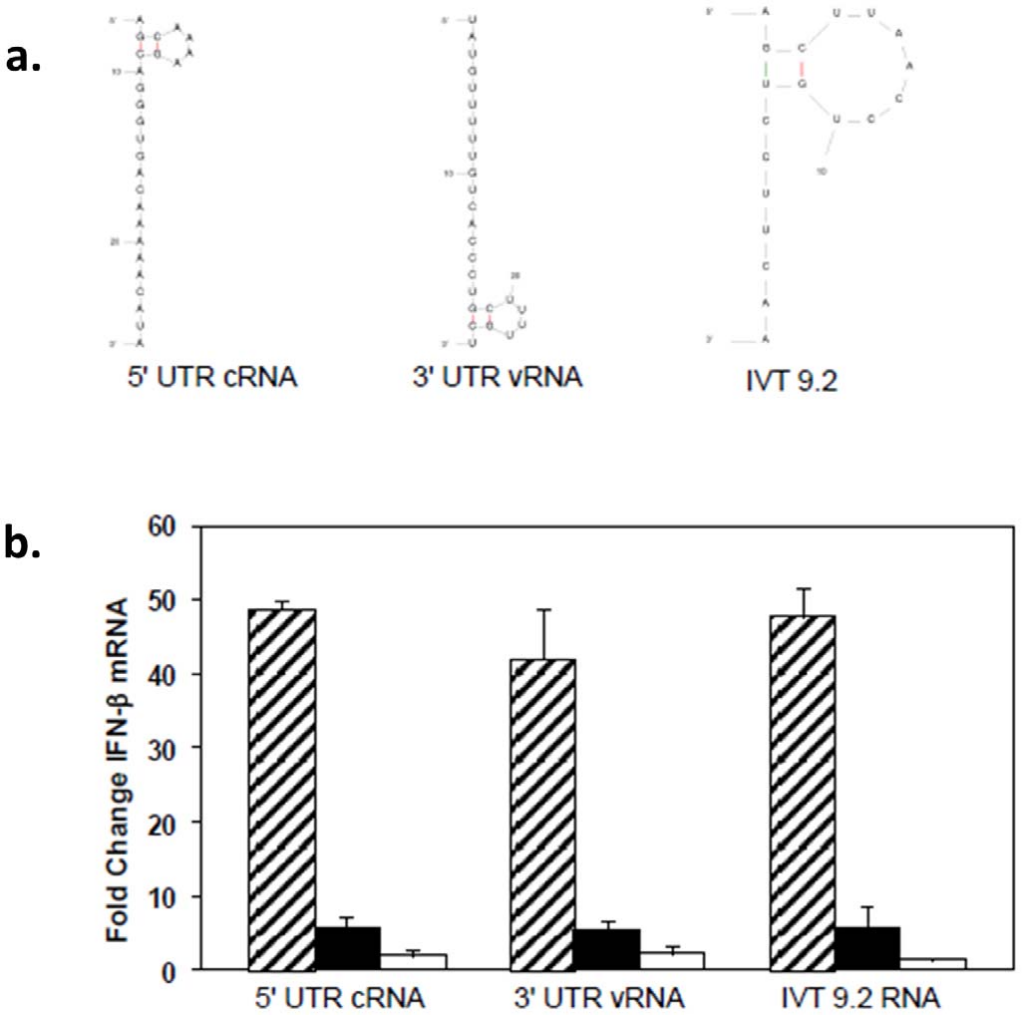
5′ PPP-independent induction of IFN-β by small influenza-derived RNA sequences. (A) The secondary structures of the IVT RNAs were predicted using the program mfold (v3.2). (B) A549 cells were transfected with 3 µg of *in vitro* transcribed RNAs from the 5′ end of the cRNA/3′ end of the vRNA sequence of NS1 gene. 24 hr post-transfection, RNA was extracted to determine the levels of IFN-β by qRTPCR. The data are shown as folds over the mock control. Hatched bar, filled bar and empty bars represent untreated, CIP-treated and capped RNAs.

After transfection into A549 cells, two sequences from the 5′ end of the cRNA (5′UTR cRNA and its complement, 3′UTR vRNA) efficiently induced IFN-β message (Fig. 2a and b) at levels comparable to a previously reported sequence, IVT 9.2 RNA, which serves as a positive control in our experiments [20], [21]. As expected, the stimulatory activities of these RNAs were highly dependent on 5′PPP, since the levels of IFN-β mRNA were 10–20 fold lower in cells transfected with CIP-treated or capped RNA products. The nucleotide compositions of these two sequences are distinct and complementary and the predicted secondary structures depict two stem loops (SL) with 2 base pair (bp) stems and 4 base loops on opposite ends of the molecules. Despite these differences, both these RNAs induced similar levels of IFN- β mRNA.

Since previous reports suggest that U/A-rich sequences are important for RIG-I recognition of RNA [15], mutations were introduced into two stretches of A nucleotides that are found within the 5′UTR cRNA (Figure S1a). The Loop Mutant and Tail Mutant had very similar total nucleotide compositions (differing by only a single A to G substitution) and identical SL structures. They also differed from the parental construct in sequence and nucleotide composition but had the same secondary structure. In spite of these similarities, the Loop Mutant lost the ability to activate IFN-β whereas the Tail Mutant did not. Loss of activity was also seen in both Loop and Tail Mutants A and B which lacked the A nucleotides in the loop structure (Figure S1b and 1c). These results suggested that A residues in the loop, but not in the tail, were important for signaling. Thus, it appeared that although RIG-I recognized ligands with diverse sequences, for a given ligand, internal sequence motifs were important.

### 5′PPP-independent interferon induction

**Figure 3:**
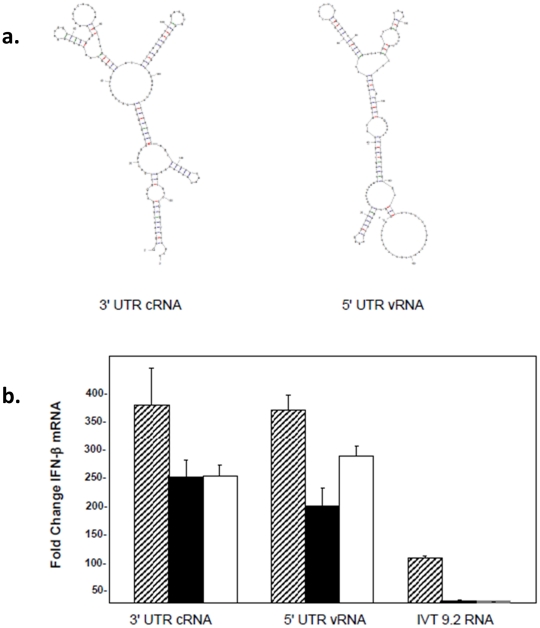
Induction of IFN-β message is triphosphate independent. (A) The secondary structures of the IVT-RNAs shown were predicted by the program mfold (v3.2). (B) A549 cells were transfected with 3 µg of UTR RNA from the 3′ end of the cRNA or 5′ end of the vRNA and RNA was isolated 24 hr post-transfection to determine IFN-β levels by qRT-PCR. The data are shown as fold increases over levels in mock transfected cells. Error bars represent the standard deviation of triplicate qRT-PCR runs using RNAs from one of three representative experiments. Hatched bar, filled bar and empty bars represent untreated, CIP-treated and capped RNAs.

**Figure 4:**
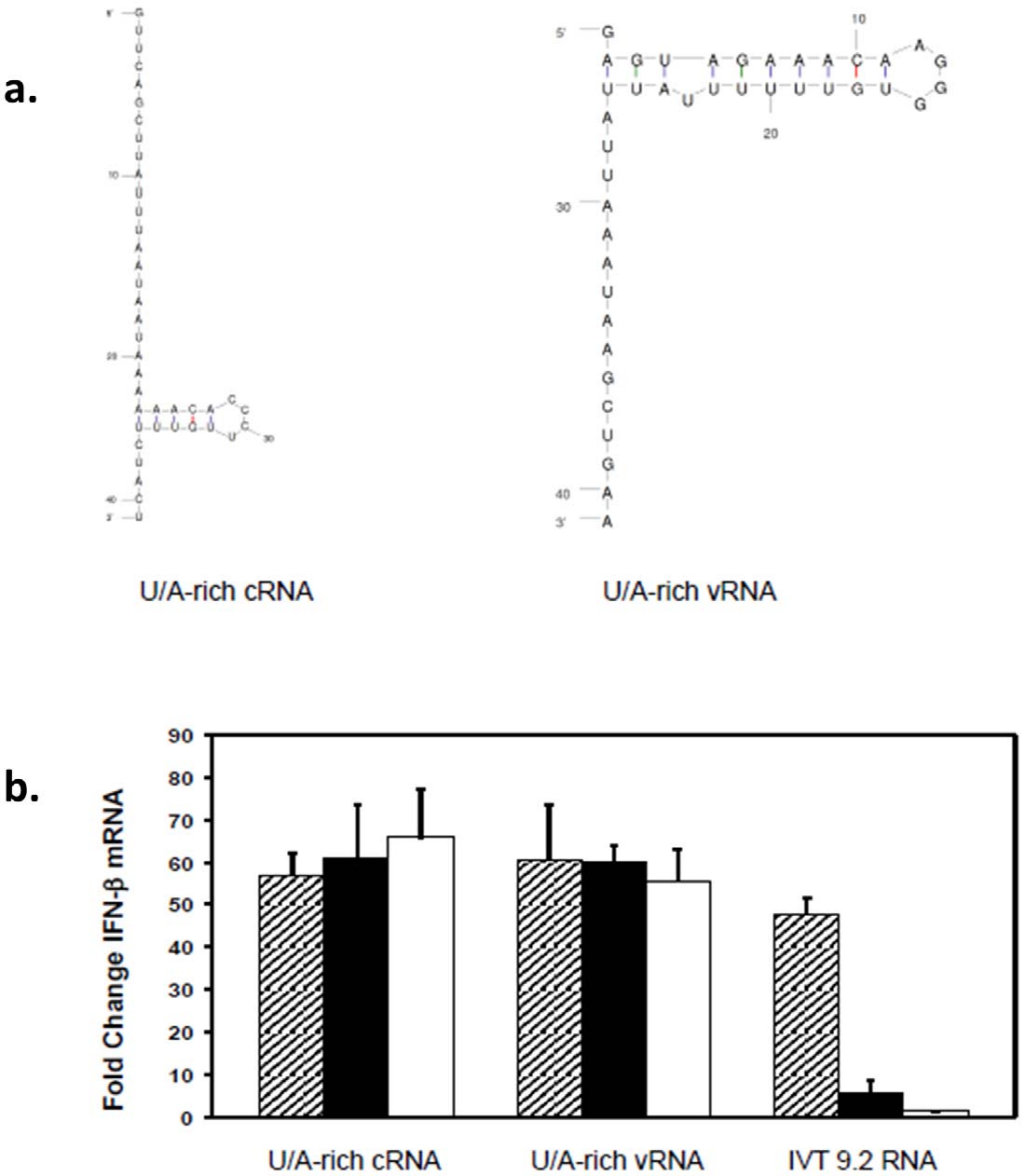
Smaller U/A rich IVT RNAs from the cRNA and vRNA UTRs are also triphosphate independent. (A) The secondary structures of the IVT-RNAs used are presented. (B) A549 cells were transfected with U/A-rich cRNA and vRNA. 24 hours post-transfection, RNA was isolated to determine IFN-β mRNA levels by qRT-PCR. The data are shown as fold increases over levels in mock transfected cells. Hatched bar, filled bar and empty bars represent untreated, CIP-treated and capped RNAs. Error bars represent the standard deviation of triplicate qRT-PCR runs using RNAs from one of three representative experiments.

In contrast to all of the shorter 5′UTR RNAs, sequences derived from the larger 3′ UTR cRNA of NS1 (172 nt) and its complementary sequence (5′ UTR vRNA) resulted in only a partial decrease in IFN-β mRNA levels in the absence of the 5′PPP, exhibiting levels that were still much greater than the positive control IVT 9.2 RNA with a triphosphate (Fig. 3a and b). Interestingly, the longer 172 nt RNA gave up to 3-fold greater induction of IFN-β mRNA than the positive control in contrast to the shorter RNA species which induced IFN-β to levels similar to the positive control (Fig. 3b vs Fig. 2b). Since these constructs were large, we focused on the terminal U/A-rich region in the context of both the cRNA and vRNA (Fig. 4a). Surprisingly, the smaller U/A-rich constructs showed complete 5′PPP independence of IFN-β mRNA induction (Fig. 4b). Like the 5′ cRNA sequences, these two 3′ cRNA sequences differed from each other in nucleotide composition and loop position. They also differed from the 5′ cRNA sequences in that they contained a much higher percentage of U and A residues (∼75% vs. ∼50%). This sequence feature has previously been identified with RIG-I PAMPs but not with the ability to signal in the absence of 5′PPP [15].

**Figure 5:**
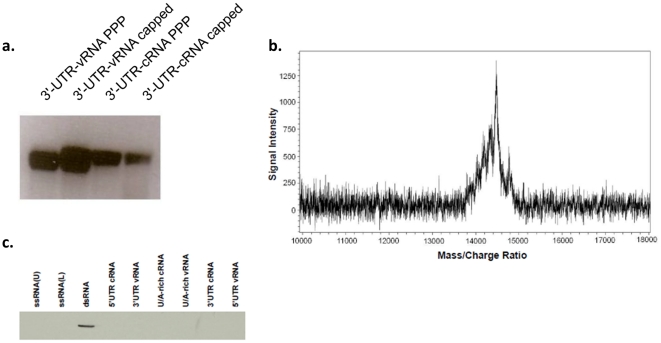
Homogeneity of IVT RNAs. (A) Denaturing agarose gels of *in vitro* transcribed RNAs show products running as single bands. (B) U/A-rich vRNA was prepared by IVT and subjected to mass determination by MALDI-TOF mass spectroscopy. A single peak was observed spanning ∼1 kDa (13.8 k–14.8 k) and corresponding to the expected mass +/− ∼1–3 nucleotides. (C) To determine if the *in vitro* transcribed RNAs are ssRNA or ds NA, RNA samples were resolved on TAE PAGE, transferred onto nylon membrane and probed for dsRNA using dsRNA specific antibodies as described in Material and Methods. None of the *in vitro* transcribed RNAs nor the 41-nt long chemically synthesized ssRNA complementary strands were detected by the dsRNA-specific antibody. Only annealed 41 bp dsRNA was detected by the dsRNA-specific antibody.

This surprising result led us to investigate the nature of our RIG-I ligands in more detail. Recent reports have indicated that IVT can result in the addition of non-templated complementary bases at the 3′ ends of transcripts [22], [23], although the efficiency of this copy-back mechanism appears to vary by template [24]. Visualization of templates used in this study by agarose gel electrophoresis showed primarily single bands (Fig. 5a). More sensitive examination of the IVT-produced U/A-rich vRNA by MALDI-TOF mass spectrometry indicated the presence of species that differed in size by approximately 1–3 nucleotides but no evidence of more extensive duplication (Fig. 5b). As a third approach to determine whether or not the single-stranded IVT RNAs contained base-paired regions, each of the six influenza sequences used above were resolved on acrylamide gels and transferred to a nitrocellulose membrane. After blotting with a dsRNA-specific antibody, only the double stranded control RNA was recognized, indicating that any base pairing of the IVT-produced RNAs was minimal and below the limits of detection of the antibody. (Fig. 5c).

Based on the sequence dependence seen for the 5′UTR cRNA, we performed mutational analysis on the U/A-rich cRNA and vRNA sequences and evaluated the consequences for IFN-β mRNA production. We substituted either all U nucleotides, or just a stretch of 6 conserved U residues, with C residues. The substitutions and their effect on the predicted RNA secondary structure is shown in Figure S2a. These changes did not eliminate IFN-β mRNA induction by RNAs containing a triphosphate as was seen with the mutations in the context of the 5′ UTR of the cRNA (Figure S2b). However, the mutated RNAs lost their ability to induce IFN-β mRNA transcription independent of the 5′PPP (Figure S2c).

### 5′PPP-independent activation of RIG-I

**Figure 6:**
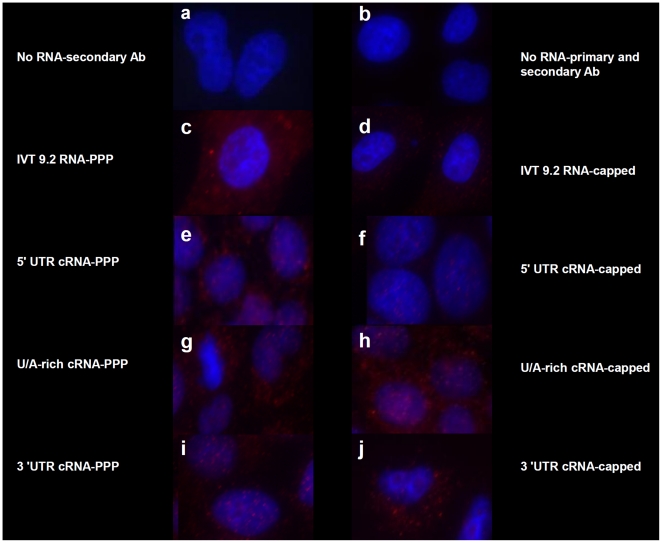
5′ PPP-independent activation of RIG-I. A549 cells were transfected with 1 µg of the indicated IVT RNAs as in Figure 2. After 24 hrs, the cells were fixed with 4% paraformaldehyde and permeabilized with a 0.2% saponin/0.1% BSA/PBS buffer. Cells were blocked using CAS overnight and probed with a conformational dependent rabbit RIG-I polyclonal primary antibody that detects the RNA-bound form of RIG-I in the cytosol and followed by staining with AlexaFluor goat anti-rabbit 549 (stains red) and Hoechst 33342 (stains nucleus blue). Cells were visualized using a Zeiss fluorescent microscope with an axiocam HRM apotome attachment using AxioVision software.

**Figure 7:**
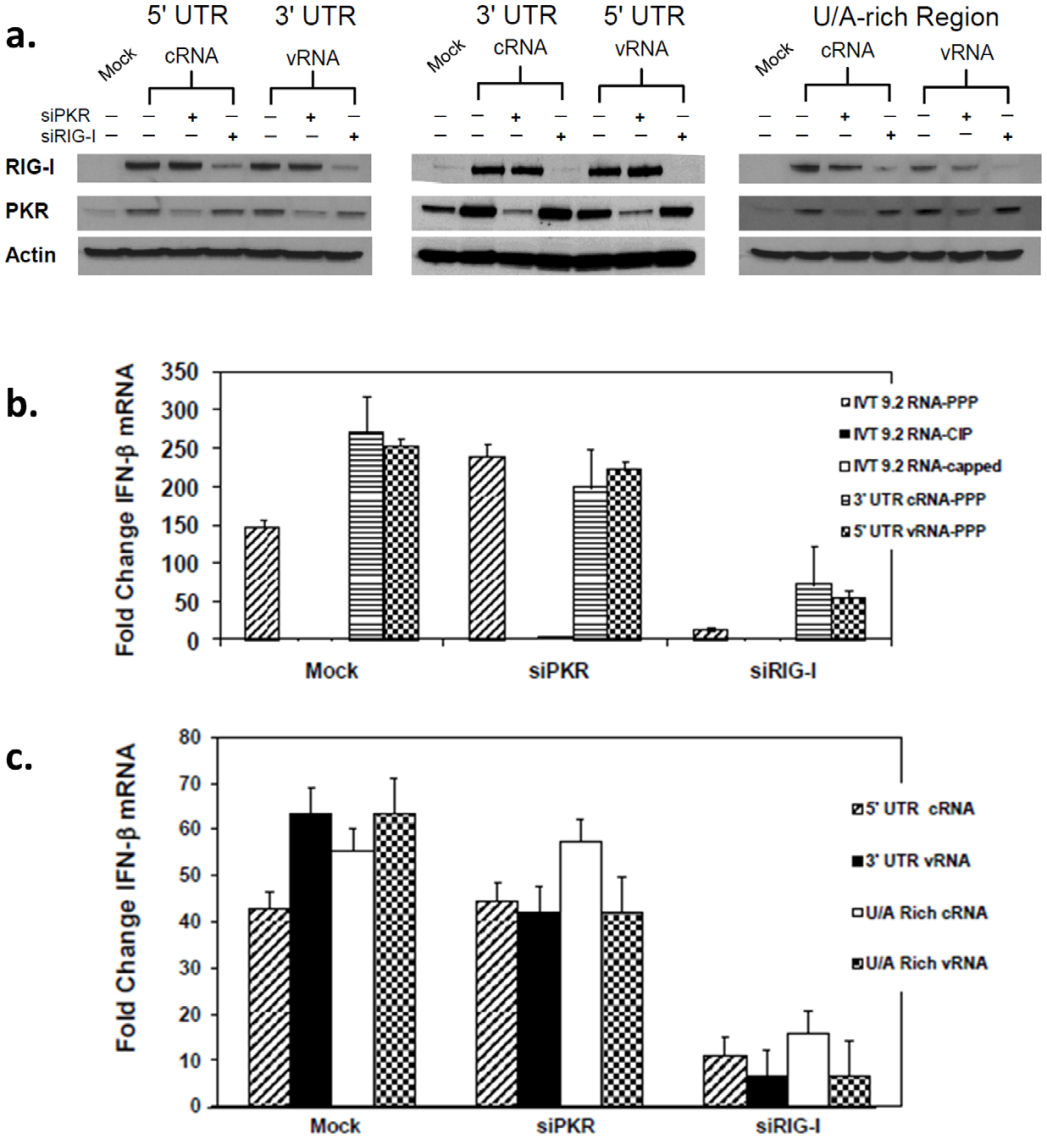
Induction of IFN and IFN-stimulated genes is inhibited by reduction of RIG-I but not PKR. A549cells were first transfected with the siRNAs against RIG-I or PKR(3 µg)as shown or mock transfected. 24 hr later, the cells were transfected again with the indicated IVT RNAs (3 µg) and processed 24 hr later for RNA as well as protein. Protein lysates are used to determine the levels of PKR and RIG-I proteins by western blot analyses and RNA is used to determine the levels of mRNA for IFN-β. (A) Western blots using the indicated antibodies of protein extracts from treated cells are shown. (B) and (C) IFN-β mRNA levels were measured using qRT-PCR. Error bars represent the standard deviation of triplicate qRT-PCR runs using RNAs from one of three representative experiments.

To ensure that IFN-β gene induction of these RNAs was mediated through RIG-I, we used two different approaches. First, we used a conformational dependent antibody that only recognized the RNA-bound form of RIG-I (T. Fujita, unpublished data). 24 hours after transfection of the appropriate RNAs, cells were fixed and stained. Although all cells expressing constructs containing a triphosphorylated 5′ end contained RNA-bound RIG-I (Fig. 6, Panels c, e, g, and i), only constructs from the 3′ cRNA UTR were also able to bind RIG-I in the absence of the 5′PPP moiety (Fig. 6, Panels h and j). These findings were confirmed by siRNA-mediated silencing of RIG-I. Because RIG-I and PKR are interferon stimulated genes, protein levels are relatively low in untreated cells, compared with transfected cells (Fig. 7a). After transfection with any of the IFN-β-inducing constructs from Fig. 2, increased levels of each of these proteins was observed. Pretreatment of cells with siRNAs directed against either PKR or RIG-I effectively reduced, but did not completely inhibit the levels of their respective target but did not substantially affect the amount of the non-targeted protein (Fig. 7a). Furthermore, siRNA against RIG-I, but not PKR, reduced, but did not completely inhibit IFN-β induction by 3′UTR cRNA, 5′ UTR vRNA, and U/A rich cRNA and vRNA species (Fig. 7b and 7c). Similar results were also found when these experiments were performed using capped RNAs (Figure S3a and b). Taken together, these results suggest that the signaling cascades leading to IFN-β mRNA induction by the RNAs used in this study are initiated by RIG-I.

One possibility to explain the ability of the 5′PPP-independent ligands to continue to signal after removal of the triphosphate group is that these ligands have a higher affinity for RIG-I than 5′PPP-dependent ligands. Since previous reports have shown that removal of the 5′PPP group from many RNA species decreases binding affinity for RIG-I (25,26), it is also possible that the 5′PPP-independent ligands do not show a similar affinity loss upon capping or dephosphorylation. To test these possibilities, we first determined the binding affinity of purified full-length RIG-I and a well-characterized fluorescein-labeled RNA molecule (FL-rU15). The dissociation constant was calculated from changes in the emission of polarized light that result from RIG-I/ligand binding obtained using a Beacon 2000 system (Invitrogen). RIG-I/FL-rU15 was then mixed with varying amounts of the unlabeled RNAs of interest and binding affinities determined from the competition with the labeled ligand (Table 1).

**Table 1:** K_d_ (nM) of RIG-I to RNA from NS1.

NS1-vRNA	5′-PPP	capped
3′-UTR (27 nt)	90±30	**160±38**
U/A-rich (41 nt)	31±5	119±12
5′-UTR (172 nt)	32±8	73±30

NS1-cRNA	5′-PPP	capped
5′-UTR (27 nt)	52±11	**187±50**
U/A-rich (41 nt)	56±9	93±26
3′-UTR (172 nt)	26±4	88±21

Affinities were measured using a fluorescence polarization assay. Values represent the mean ± standard deviation of at least two independent experiments. The RNA molecules with the lowest affinities (in bold) neither bound RIG-I *in vivo* (Fig. 6) nor induced IFN expression (Fig. 2).

Although the binding affinities of the 5′PPP-independent vRNAs (U/A-rich and 5′UTR) are higher than the affinity of the 5′PPP-dependent 3′UTR vRNA (∼30 nM vs 90 nM, see Table 1), this relationship does not hold for the cRNAs. Thus a universal higher affinity for the independent vs dependent ligands is not observed. Likewise, the second possibility noted above is also not supported since a decrease in binding affinity accompanied the loss of the 5′PPP group for all RNAs tested, including the U/A-rich and constructs that still fully activated RIG-I when the 5′PPP was removed. However, it should be noted that the affinities of the 5′PPP-independent ligands remained relatively high suggesting that the residual affinity, likely mediated by the U/A-rich regions, remained sufficient for productive RIG-I interaction and activation.

## Discussion

To date, several structural studies have examined the interface between the C-terminal domain of RIG-I with and without bound RNA ligands [25], [26], [27], [28]. The most convincing data suggest that the primary contacts are between conserved residues in the protein and both the terminal 5′PPP group and the phosphodiester backbone near the 5′ end of the RNA molecule rather than the nitrogenous bases. Thus, it is not readily apparent from the structures how to explain the observed sequence dependence seen here. One possibility is that other regions of RIG-I that contact the ligand, such as the helicase domain, do so in a sequence-dependent manner. However, at this time, no structures for the complete RIG-I molecule are available to confirm or refute this. A second possibility is that different ligand sequences do not affect RIG-I binding directly, but instead modify structural rearrangements or biochemical activities of RIG-I, such as ATPase or helicase activity, that are required for full signaling capabilities. Additional studies into the exact nature of the sequence requirements for each of these features of RIG-I activation will be required to distinguish between these and other hypotheses.

By examining the untranslated regions from the influenza genome that are highly conserved, both among different influenza viruses and genome segments of a single virus we have identified several sequences capable of activating RIG-I and inducing IFN-β transcription. These ligands vary by both sequence and predicted structure and illustrate the promiscuity of the RIG-I sensor, consistent with its ability to target a wide variety of viral infections. For these specific examples, we have also identified required sequence motifs that contribute to the ability of these ligands to interact with RIG-I in the absence of the normally critical 5′PPP group. Further investigation of this phenomenon is likely to increase the efficiency and ease of production and decrease the costs associated with designing synthetic RIG-I-based antiviral therapies.

## Materials and Methods

### Cell lines

Human lung epithelial cells (A549) were grown in DMEM (Life Technologies) supplemented with 10% FBS, 100 units/mL penicillin, and 100 µg/mL streptomycin.

### Preparation of RNA

*In vitro* transcribed RNA was prepared using the Ambion MEGAscript T7 High Yield Transcription kit according to the manufacturer's instructions. Templates were prepared by annealing complementary DNA oligos containing a T7 promoter followed by the desired target sequence. Transcription reactions proceeded for 4–16 hrs with no difference in biological activity. Following the reaction, the DNA template was digested with DNase I (NEB, Ipswitch, MA) and the RNA purified and isolated using TRIzol (Invitrogen, Carlsbad, CA), followed by ethanol precipitation. Capped RNA was produced either by replacing the GTP in the transcription reaction with a 12∶1 ratio of m7G(5′)PPP(5′)G cap analog:GTP or by using the ScriptCap m^7^G Capping System (EPICENTRE Biotechnologies, Madison, WI) according to the manufacturer's instructions. CIP-ssRNA was made by removing the functional 5′PPP end with calf intestinal alkaline phosphatase (CIP, NEB) treatment.

### Functional analysis

For transfections of A549 cells, 3 µg of RNA was used to transfect each well of a 6 well tissue culture plate using Lipofectamine 2000 (Invitrogen) as the transfection reagent. At designated time points, protein and RNA were harvested from duplicate wells for Western and qRT-PCR analyses. Total protein was separated on a 4–15% SDS-PAGE gel and the separated proteins were transferred to a nitrocellulose membrane. Western blots were performed using commercial antibodies purchased from Sigma (actin), Santa Cruz Biotechnology (RIG-I,), and Cell Signaling (PKR). The relative amount of intracellular RNA for each gene of interest was quantified by qRT-PCR on a Stratagene Mx3000P (Stratagene, La Jolla, CA.), using the Superscript III Platinum SYBR Green One-Step qRT-PCR kit (Invitrogen) according to the manufacture's protocol, and expressed as a fold change. Primers used are available upon request.

### RNA secondary structure prediction

Secondary structures were predicted using the RNA secondary structure predicting program, mfold v3.2 [19].

### Microscopy

A549 (1×10^4^) cells were plated on Nunc LabTek II (ThermoFisher, Rochester, NY) chambered slide flasks in DMEM with 10%FBS, penicillin-streptomycin, and L-Glutamine. A549 cells were transfected with one µg of each RNA species using Lipofectamine 2000 (Invitrogen, Carlsbad, CA). Cells were fixed 24 hours post-transfection using 4% paraformaldehyde and permeabilized using a 0.2% saponin/0.1% BSA/PBS buffer. Cells were blocked using CAS block (Invitrogen) overnight and probed with a conformational dependent RIG-I polyclonal antibody generated by Dr. Fujita, AlexaFluor goat anti-rabbit 549, and Hoechst 33342 (Invitrogen). Cells were visualized using a Zeiss fluorescent microscope with an axiocam HRM apotome attachment using AxioVision software (Carl Zeiss, USA).

### RNA competition assay

Binding of RIG-I to labeled RNA was measured by a change in polarization (ΔmP) on a Beacon 2000 fluorescence polarization system (Invitrogen). RIG-I (0–1000 nM) and 0.1 nM 3′-fluorescein-labeled rU15 (FL-rU15) were mixed in binding reaction buffer (50 mM HEPES, pH 7.5, 50 mM NaCl, 5 mM MgCl_2_ and 0.5 mM Tris[2-carboxyethyl] phosphine) and incubated for 30 s at 25°C. Experiments were performed in reduced light and data were plotted using KaleidaGraph (Synergy Software).

In RNA competition assays, 0.1 nM FL-rU15 was pre-incubated briefly with 100 nM RIG-I in 50 mM HEPES, pH 7.5, 100 mM NaCl, 5 mM MgCl_2_ and 10 mM BME at room temperature (final volume was 100 µl), and then 10–1000 nM of various competitor RNAs was added. The solution was incubated at 25°C for 30 s before each measurement.

### Characterization of in vitro transcribed RNA

*In vitro* transcribed RNA samples (30 µg) were resolved on a 5% acrylamide gel at 90 mA for 2 hr in TAE buffer in the presence of RNase inhibitor (RNasin; Promega, USA) (10 U/ml). A 41-nt long chemically synthesized ssRNA (complementary strands) and its annealed 41 bp dsRNA product were used as controls. RNA was transferred to nylon membrane using electrophoretic transfer apparatus. The nucleic acids were not denatured prior to or after the transfer. After the transfer was complete, RNAs on the membrane was cross-linked by Stratagene cross-linker. Membrane was blocked using 5% BSA in TBS in the presence of RNase inhibitor for 3 hr and incubated with anti-dsRNA antibodies (http://www.engscicons.de/monoclonal2005_eng/J2_desc2005.htm) (2 µg/ml) overnight at 4 C. Antibody signals were detected by chemiluminescence using secondary antibodies conjugated to horseradish peroxidase and an ECL detection kit (Amersham Biosciences, Inc., NJ, USA).

MALDI-TOF-MS of IVT RNA was performed using an Ultraflex III mass spectrometer (Bruker Daltonics, Billarica, MA) operated in positive linear mode. The matrix was made up of 1∶1 (v/v) mixture of 15 mg/ml of ammonium citrate in 0.1% trifluoroacetic acid/30% acetonitrile/water and 80 mg/ml of 2′,4′,6′-trihydroxyacetophenone monohydrate in ethanol. The sample was mixed 1∶8 with the matrix and analyzed with external calibration using synthetic oligodeoxyribonucleotides.

## Supporting Information

Figure S1 **Induction of IFN-β message by IVT RNAs is sequence dependent.** (A) The secondary structures of the 5′ UTR cRNA RNAs after base substitutions within the A rich regions were predicted using mfold (v3.2). (B) and (C) A549 cells were transfected with the RNA constructs shown and RNA was isolated 24 hr post-transfection. IFN-β mRNA levels were quantified using qRT-PCR. Error bars represent the standard deviation of triplicate qRT-PCR runs using RNAs from one of three representative experiments.
(TIF)

Figure S2 **5′PPP-independent induction of IFN-β message is sequence dependent.** (A) The secondary structures of the U/A-rich regions or mutant RNAs after base substitutions as predicted by the program mfold (v3.2) are shown. (B) and (C) A549 cells were transfected with the indicated IVT RNAs and RNA was isolated 24 hr post-transfection to quantitate IFN-β message by qRT-PCR. Error bars represent the standard deviation of triplicate qRT-PCR runs using RNAs from one of three representative experiments.
(TIF)

Figure S3 **Induction of IFN and IFN-stimulated genes is inhibited by reduction of RIG-I but not PKR using IVT capped RNAs**. (A) A549 cells were transfected with the siRNAs shown or mock transfected. 24 hr later, cells were transfected again with the indicated IVT RNAs and the cells were processed 24 hr post-secondary transfection. (B) Protein lystaes were used to determine the levels of RIG-I and PKR by western blot analysis. (C) RNA isolated from A549 cells was used to measure IFN-β mRNA levels by qRT-PCR. Error bars represent the standard deviation of triplicate qRT-PCR runs using RNAs from one of three representative experiments.
(TIF)
