# Supplementary figures and images for: The 3′ Untranslated Regions of Influenza Genomic Sequences Are 5′PPP-Independent Ligands for RIG-I
Source: PLoS One. 2012 Mar 15;7(3):e32661. doi: 10.1371/journal.pone.0032661 (PMC3305289; doi:10.1371/journal.pone.0032661)

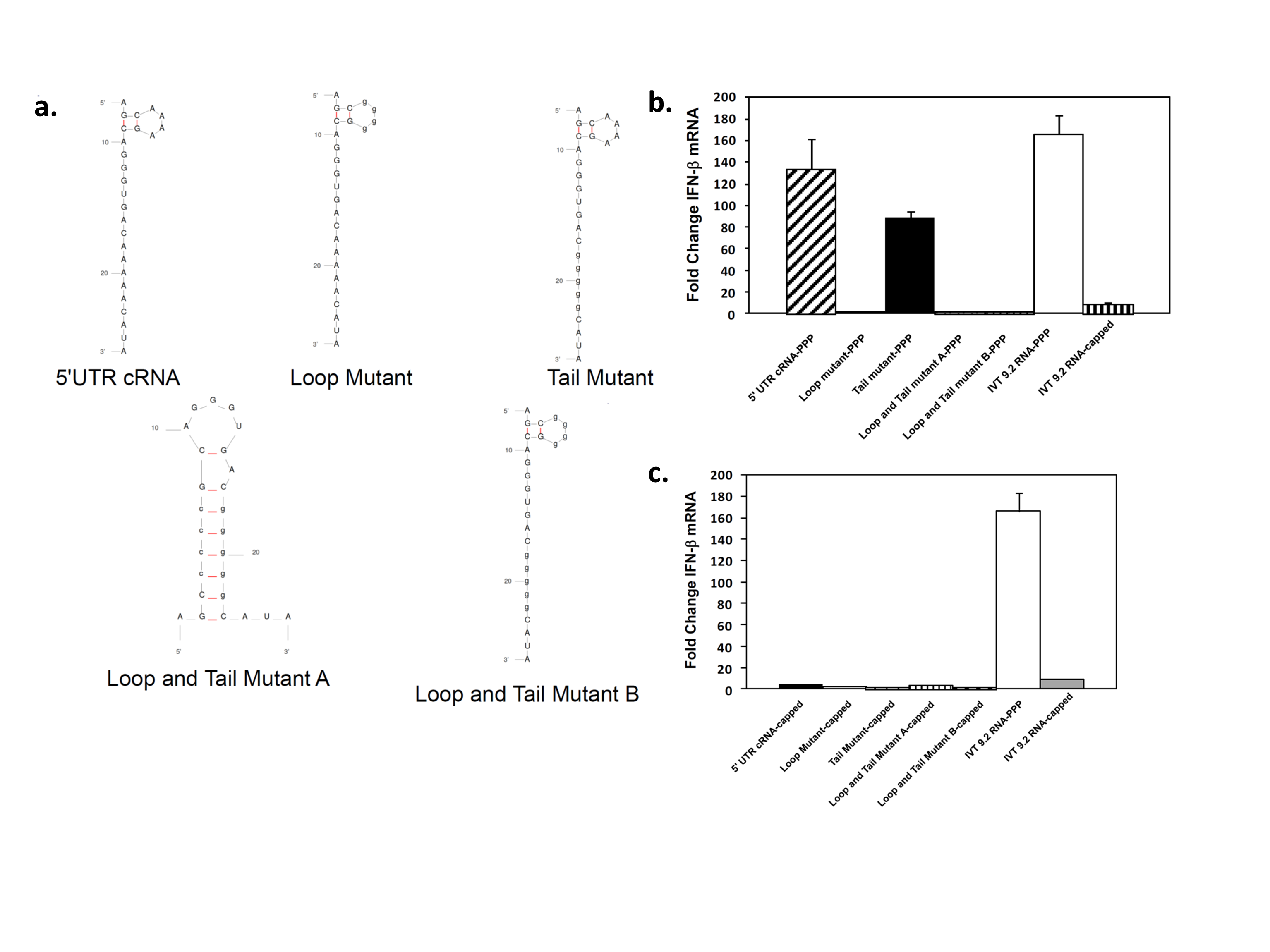

Supplement: Figure S1 — Induction of IFN-β message by IVT RNAs is sequence dependent. (A) The secondary structures of the 5′ UTR cRNA RNAs after base substitutions within the A rich regions were predicted using mfold (v3.2). (B) and (C) A549 cells were transfected with the RNA constructs shown and RNA was isolated 24 hr post-transfection. IFN-β mRNA levels were quantified using qRT-PCR. Error bars represent the standard deviation of triplicate qRT-PCR runs using RNAs from one of three representative experiments. (TIF) [file pone.0032661.s001.tif]

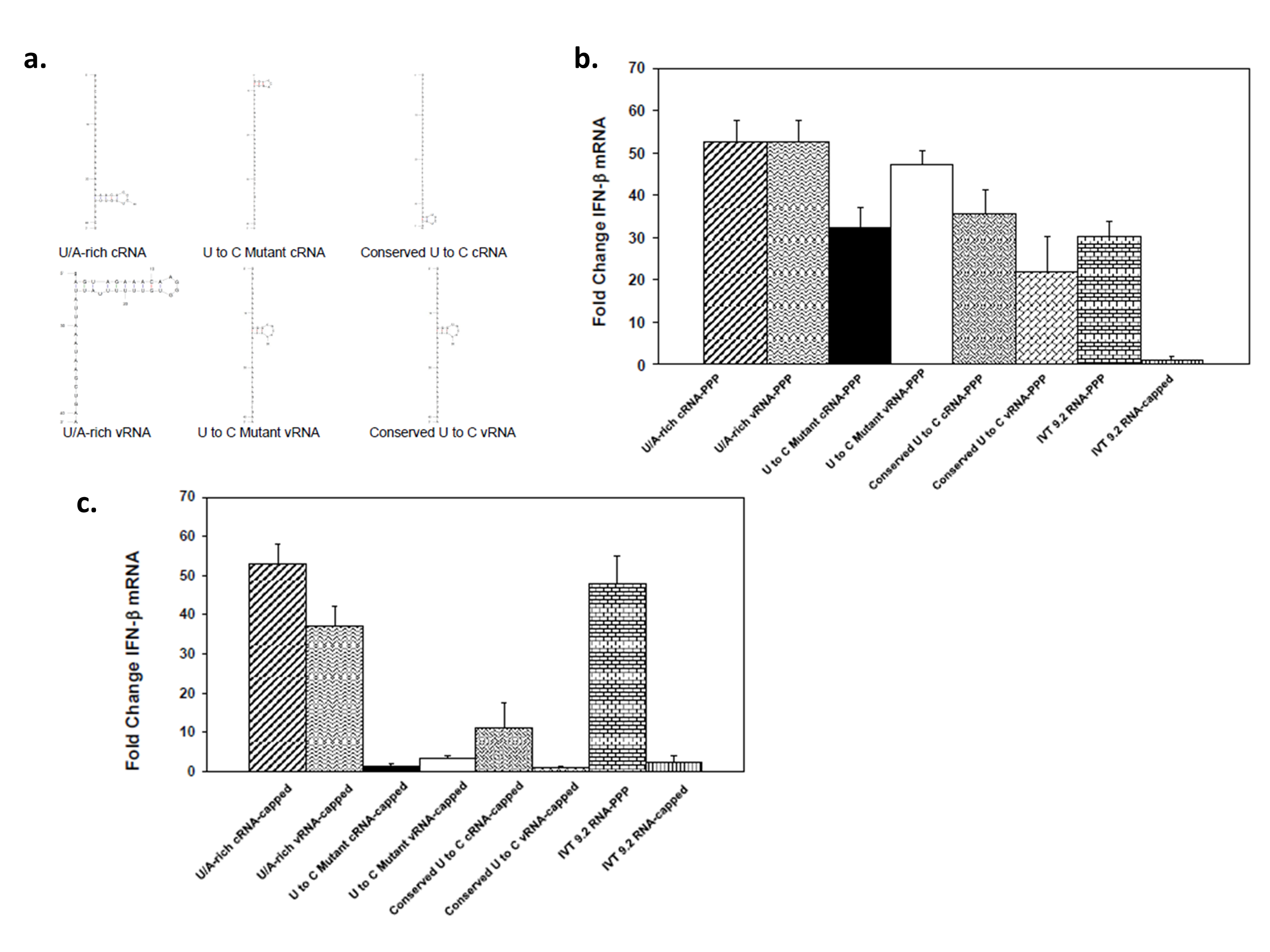

Supplement: Figure S2 — 5′PPP-independent induction of IFN-β message is sequence dependent. (A) The secondary structures of the U/A-rich regions or mutant RNAs after base substitutions as predicted by the program mfold (v3.2) are shown. (B) and (C) A549 cells were transfected with the indicated IVT RNAs and RNA was isolated 24 hr post-transfection to quantitate IFN-β message by qRT-PCR. Error bars represent the standard deviation of triplicate qRT-PCR runs using RNAs from one of three representative experiments. (TIF) [file pone.0032661.s002.tif]

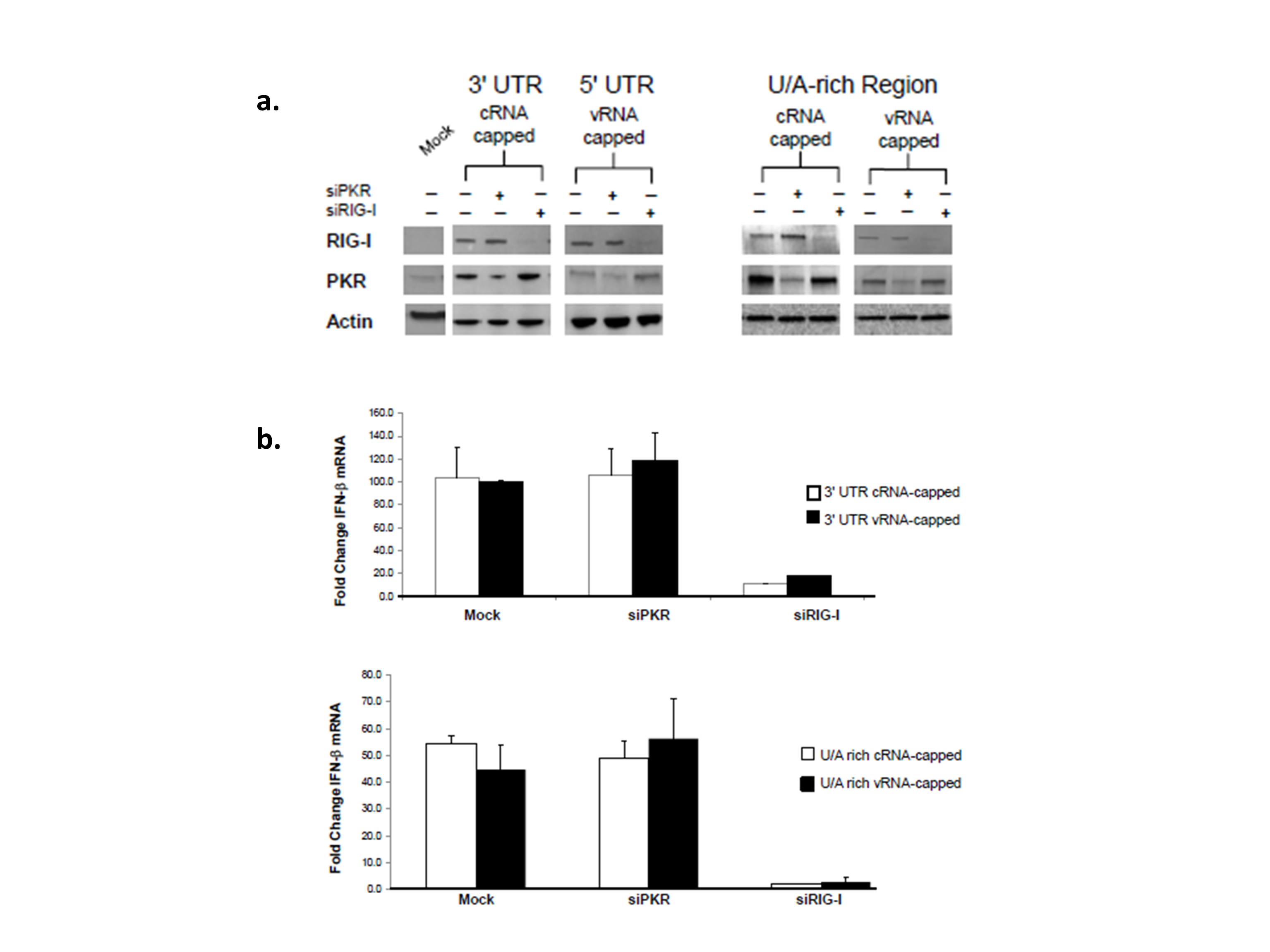

Supplement: Figure S3 — Induction of IFN and IFN-stimulated genes is inhibited by reduction of RIG-I but not PKR using IVT capped RNAs. (A) A549 cells were transfected with the siRNAs shown or mock transfected. 24 hr later, cells were transfected again with the indicated IVT RNAs and the cells were processed 24 hr post-secondary transfection. (B) Protein lystaes were used to determine the levels of RIG-I and PKR by western blot analysis. (C) RNA isolated from A549 cells was used to measure IFN-β mRNA levels by qRT-PCR. Error bars represent the standard deviation of triplicate qRT-PCR runs using RNAs from one of three representative experiments. (TIF) [file pone.0032661.s003.tif]
